# Editorial: New green extraction methods for the sustainable recovery of functional plant secondary metabolites

**DOI:** 10.3389/fpls.2023.1186180

**Published:** 2023-04-04

**Authors:** Maura Ferri, Óscar Rodríguez, Annalisa Tassoni

**Affiliations:** ^1^Department of Biological, Geological and Environmental Sciences, University of Bologna, Bologna, Italy; ^2^IRIS Technology Solutions, SL., Cornellà de Llobregat, Spain

**Keywords:** bioactive compounds, functional secondary metabolites, phytochemical bioactivities, green extraction techniques, phytochemical recovery

Secondary metabolites are small molecules synthesized by plants to make them adaptive and competitive in their own environment. More than 200,000 phytochemicals are known to display a wide range of effects on living organisms, including plants, animals and humans. In this context, food, nutraceutical, pharmaceutical, cosmetic, agricultural and new material industries are actively seeking for new plant sources to recover active compounds while aiming at sustainable natural resource utilization and protection of the environment. New green extraction techniques are being developed to substitute the standard not-environmentally friendly chemical and solvent-based methodologies. Several possibilities have been explored, such as natural deep eutectic solvents (NADES), power ultrasound or microwave radiation, enzymatic hydrolysis, supercritical or pressurized liquid extractions. Depending on the final aim, the applied methods can lead to total phytochemical extract or to the selective recovery of specific classes of plant-derived compounds. Yield and extraction specificity are usually among the most important parameters considered. The interference of molecules co-extracted with the target compounds should be minimized, as well as degradation of target compounds or artifacts formation, while the techno-functional features of the targeted molecules must be ensured both during and after extraction.

This Research Topic contributes to the field of green extraction of phytochemicals and comprises a Mini Review, two Original Research papers and one Methods paper.

Within this Research Topic, Li reviewed the physicochemical properties of NADES and their application in phytonutrient recovery. NADES are considered the new frontier of green extractions thanks to their environmental friendliness, easy biodegradability, and low cost. From a chemical point of view, NADES have high solvency capacity and low volatility. They are composed of two components from natural sources, one hydrogen bond acceptor (HBA) and one hydrogen bond donor (HBD), mixed by a simple preparation procedure. NADES are also easily designable, due to the possibility of using different HBA and HBD combinations, as reported by Li. Moreover, NADES extracts have good biocompatibility and the recovered molecules have generally high solubility, stability and bioavailability. Li’s Mini Review also summarized several applications within plant nutrients different extracts.

In addition to the above mentioned green and useful characteristics, the sustainability of NADES extraction of phytochemicals, can be enhanced by using residues or by-products as initial raw material. García-Roldán et al. studied the valorisation of spent coffee ground *via* NADES extraction of polyphenols. The coffee processing by-product was treated by hydrated betaine:triethylene glycol or choline chloride:1,2-propanediol (1:2 molar ratio) and, for comparison, by hot water or hydroalcoholic solution. Total phenolic, protein and reducing sugar contents were quantified for the obtained extracts. HPLC-UV/Vis qualitative analyses were also performed, and antioxidant and antimicrobial activities were assayed. NADES were as effective as conventional solvents in the extraction of polyphenols (e.g., 3-O-caffeoylquinic, gallic and caffeic acids) with the added advantages of operating at milder temperature conditions and by using eco-friendly compounds. Moreover, extracts exerted an antimicrobial activity that was 10 times higher than obtained using the ethanolic and aqueous solvents. The authors concluded that NADES extracts could be used as formulation aid for food ingredients.

Sustainable extraction approaches are particularly important when the demand of specific phytochemicals cannot be supported by standard extraction processes. Arya et al. reported the case of nard (*Nardostachys jatamansi* (D. Don) DC), a highly valued medicinal herb. Nard has been largely used in traditional medicine, and the increasing demand cannot be satisfied by the conventional extraction methodologies. The authors presented microwave-assisted extraction (MAE) as an alternative green approach to recovery of functional secondary metabolites from plant. Key process parameters (irradiation temperature and time, microwave power) were optimized by using Box Behkhen Design (BBD). Treated plat materials were studied using scanning electron microscopy and the effect of radiations was highlighted. The extracts obtained by MAE and by traditional maceration were assessed for the recovery of secondary metabolites and anti-Alzheimer’s potential. The yields of total phenols and total flavonoids were significantly enhanced by MAE, as well as the concentration of various sesquiterpenes and steroidal compounds. In agreement with composition results, a significant improvement in anti-Alzheimer’s potential was also observed in mice following administration of optimized MAE extract. According to the reported findings, authors concluded that the MAE could be a successful and green alternative for the recovery of secondary bioactive metabolites from nard.

To obtain a plant extract is usually not sufficient for the valorisation of phytochemicals. Extract characterization is also a fundamental step and new methods for identification and quantification of all components of such complex mixtures must be improved and validated. In the present Research Topic, the Methods article by Petibon and Wiesenberg described the simultaneous extraction and characterization of leaf chlorophylls, carotenoids and their derivatives (including isomers). The study aimed at more exhaustively characterizing pigments contributing to the leaf optical properties in the visible range of the light spectrum to better calibrate the commonly used optical sensors. A sequential extraction and liquid chromatography-diode array detection approach (with the newest generation of core–shell columns) was developed, optimized, and validated with spinach leaves. The method was then successfully performed on leaf pigment extracts of beech (*Fagus sylvatica* L.), chosen as a model of a European deciduous tree species. According to the authors’ conclusions, obtaining the fingerprint of leaf pigments could be useful in studies of forest tree physiology and for ecophysiological applications. The new method allowed a precise identification of a wide range of pigments and their derivatives, with a detection limit of a few nanograms per millilitre, leading to the detection of intraspecific variations within an individual tree over a single growing season.

In recent years, the interest in developing and improving green techniques for the extraction and valorization of plant functional secondary metabolites has largely increased. Most of the green techniques mentioned in this Research Topic are not off-the-shelf technologies. They have been proven successful at laboratory scale, but need to be developed and scaled up, while considering the unique conditions for every application. The present Research Topic collects valuable studies that contribute to this important field, paving the way for future investigations.

## Author contributions

MF wrote the article. AT and OR revised the manuscript. All authors approved the article.

